# Development and Validation of the Intimate Partner Sexual Violence Scale (IPSVS): A Multi-Dimensional Scale to Measure Sexual Violence in Intimate Relationships

**DOI:** 10.1177/08862605241287803

**Published:** 2024-10-15

**Authors:** Laura Tarzia, Cynthia Brown, Elizabeth McLindon, Kelsey Hegarty

**Affiliations:** 1 The University of Melbourne, Carlton, VIC, Australia; 2 The Royal Women’s Hospital, Parkville, VIC, Australia

**Keywords:** adult victims, sexual assault, domestic violence

## Abstract

Intimate partner sexual violence (IPSV) is defined as any non-consensual sexual behavior perpetrated within an intimate relationship. It is overwhelmingly perpetrated by men against women and causes significant harm to the health and wellbeing of victim/survivors. Although it is generally accepted that IPSV is globally prevalent, to date, comprehensive instruments to measure IPSV have been lacking. Moreover, existing measurement tools have largely failed to capture the diversity of perpetrator tactics, the deeply degrading and dehumanizing nature of many IPSV behaviors, and the role of perpetrator intent. As a result, there is a dearth of robust quantitative data to help understand the nature and magnitude of the problem. This article describes the development of a new multi-dimensional measurement tool—the Intimate Partner Sexual Violence Scale (IPSVS). The IPSVS is grounded in qualitative evidence from victim/survivors and examines IPSV as a multi-dimensional phenomenon with perpetrator intent as a central organizing principle. Following consultation with experts, a representative sample of 702 women victim/survivors in Australia completed an online survey including 34 IPSV behaviors to establish reliability and validity of the items. Exploratory factor analysis revealed 20 items across 3 factors—“Dominance and humiliation,” “Emotional coercion,” and “Aggressive indifference”—explaining 52.681% of the variance. Cronbach’s alphas ranged from .741 to .890. These domains highlight that different perpetrator motivations may underlie different types of IPSV, challenging the predominant classification of IPSV according to severity or level of physical harm. The IPSVS has the potential to dramatically improve measurement of sexual violence in intimate relationships and has important implications for future research and practice.

## Introduction

Sexual violence is a globally prevalent human rights issue, overwhelmingly perpetrated by men against women (World Health Organization, 2013). While stranger assaults tend to dominate the public consciousness, it is far more common for women to experience sexual violence at the hands of an intimate partner or ex-partner (Black et al., 2010; Cox, 2016); this phenomenon is referred to as intimate partner sexual violence (IPSV). Studies have repeatedly suggested that IPSV is associated with a range of serious physical, reproductive, and psychological harms (Potter et al., 2021; Tarzia et al., 2018). These include post-traumatic stress disorder, depression and anxiety, alcohol and substance misuse, eating disorders, unwanted pregnancies, and self-harm and suicidality (World Health Organization, 2013). Yet, despite its devastating impacts and the associated economic burden on society (Australian Institute of Health and Welfare, 2020), IPSV remains neglected and poorly addressed in research, policy, and practice (Logan et al., 2015).

A major barrier to effective IPSV prevention and response is a lack of comprehensive instruments to measure IPSV and, by extension, a dearth of robust quantitative data on the nature and magnitude of the problem (Bagwell-Gray et al., 2015; Logan et al., 2015). IPSV is an umbrella term for a broad range of non-consensual sexual acts (or attempted acts) perpetrated by a partner. Qualitative work strongly suggests a diversity of perpetrator tactics (Bergen, 1995; Tarzia, 2021a, 2021b; Tarzia & Hegarty, 2022; Russell, 1990), including persistent pressure and blackmail, manipulation, forced exposure to pornography, and non-consensual rough sex (Yardley, 2021). Yet, to date, the range of IPSV behaviors has not been reflected in the majority of cross-sectional research, with the extant literature typically reducing measurement of IPSV to threatened or forced sex (Anderson et al., 2021; Logan et al., 2015). This is highly problematic given that, in contrast to sexual violence perpetrated by a stranger, many victim/survivors do not identify their experiences of IPSV as “forced sex” (Harned, 2005; Logan et al., 2007), instead framing the issue as part of normal relationship negotiations (Basile, 2002), a deficiency with their own libido or a case of mismatched sexual needs (Tarzia, 2021c).

While there are some comprehensive measurement tools that have been developed to measure sexual violence more broadly, these do not adequately capture the context and dynamics of IPSV. For instance, the most widely used sexual violence measure, the Sexual Experiences Survey (SES) (Koss, 2007), includes several items about the use of alcohol or substances to incapacitate the victim/survivor, as well as behaviors such as unsolicited exposure of the genitals or being watched while naked. Although these behaviors can occur in the context of IPSV, qualitative work suggests that many other tactics are more common (Tarzia, 2021a, 2021b; Tarzia & Hegarty, 2022), although these are not captured by the SES. For example, coercion through inducing a sense of obligation, appealing to the victim/survivor’s emotions (e.g., “you don’t love me if you won’t have sex”), or suggesting that the victim/survivor is frigid or unhealthy if they decline sex (Tarzia, 2021c; Tarzia & Hegarty, 2022) are all absent. Although a recent attempt has been made to adapt the SES to address IPSV by distinguishing between acts occurring “with a romantic partner” and “not with a romantic partner” (Anderson et al., 2021), the content, emphasis, and focus of the questions were not altered, limiting its usefulness. Another scale, the Multidimensional Sexual Coercion Questionnaire (Raghavan et al., 2014), more effectively addresses the spectrum of sexually coercive behaviors in relationships, with seven domains covering threats and/or physical force, exploitation, humiliation/intimidation, pressure, manipulation, hopelessness, and helplessness (Raghavan et al., 2014). However, even here, the deeply degrading and dehumanizing nature of many IPSV behaviors—which is a strong theme throughout qualitative research (Tarzia, 2021a;Tarzia & Hegarty, 2022)—is not captured.

Other measures attempting to capture experiences of sexual violence in the context of an intimate relationship, such as the Sexual Coercion in Intimate Relationships Scale (Shackelford & Goetz, 2004), fail to address the physical forms of IPSV. Yet, survivors often report behaviors such as use of physical force to obtain sex, strangulation, or non-consensual rough sex. On the other hand, broader measures of intimate partner violence (IPV) such as the Conflict Tactics Scale (Straus et al., 1996) or the Severity of Violence Against Women Scale (Marshall, 1992) and the Composite Abuse Scale (Hegarty et al., 2005) only include limited questions on sexual violence. These scales have often been used to measure IPSV prevalence but tend to return far lower rates than measures focused solely on sexual violence (Anderson et al., 2021).

A major limitation of all the current instruments for measuring sexual violence in relationships is that there is a lack of focus on the intent or motivation of the perpetrator. Instead, behaviors tend to be categorized based on type or “severity.” This is a broader issue of conceptual framing, where IPSV is viewed as a continuum (Raiche et al., 2023) with verbal coercion at one end and physical force at the other. For example, Bagwell-Gray (2019) has recently developed a taxonomy of IPSV with four categories of behaviors, distinguished from each other by the degree of accompanying physical violence and invasiveness or “penetrativeness”: sexual assault, sexual coercion, physically forced sexual activity, and sexual abuse. While we do not disagree that sexual violence encompasses a spectrum of behaviors, measuring harm or grouping behaviors together based purely on the degree of physical force is inconsistent with what victim/survivors say about their experiences (Bergen, 1995; Tarzia, 2021a, 2021c; Tarzia & Hegarty, 2022; Finkelhor & Yllo, 1985; Russell, 1990). Qualitative research does not support the theory that increased physical force or even invasiveness is the only important factor distinguishing one form of IPSV from another, nor that physical force during IPSV necessarily determines the degree of emotional harm (Tarzia, 2021a; Tarzia & Hegarty, 2022). For some survivors, the sense of betrayal or the experience of sexual degradation and humiliation can be far more impactful, even without physical force or violence (Tarzia, 2021a).

Earlier qualitative work on IPSV made some forays into defining typologies that take perpetrator motivation into account. Finkelhor and Yllo (1985), for instance, distinguished between “battering rape” (hitting, humiliation) where the motivation is punishment for some perceived transgression, “force only rape” (minimum force required to ensure compliance) which is motivated by entitlement, and “obsessive rape” (mutilation, sexual sadism) where the perpetrator experiences sexual pleasure at causing their partner pain. Proulx and Beauregard (2014), on the other hand, proposed a typology that categorizes perpetrators as either angry (high aggression, dissatisfaction with sexual life); hypersexual (high levels of violence, deviant sexual fantasies, high frequency of sexual contact); or lonely (limited use of physical violence, low self-esteem). Yet, to our knowledge, neither these typologies nor perpetrator motivation in general have been considered in scale development.

Considering the abovementioned limitations, it is unsurprising that prevalence rates for IPSV have varied widely (Logan et al., 2015). Some of this variation can be accounted for by context (e.g., college samples tend to report high rates of sexual violence (Bonar et al., 2022), as do many developing countries (Bagwell-Gray et al., 2015; Logan et al., 2015; Sardinha et al., 2022)); however, it is highly likely that a large proportion of the variation is due to inconsistencies in language and conceptual understanding of IPSV. In response to these issues, this paper describes the development and validation of a new instrument—the Intimate Partner Sexual Violence Scale (IPSVS). The IPSVS is informed by robust qualitative data in the form of in-depth interviews with victim/survivors (Tarzia, 2021a, 2021b, 2021c; Tarzia & Hegarty, 2022) and examines IPSV as a multi-dimensional phenomenon with perpetrator intent as a central organizing principle.

## Methods

Our study was guided by previous scale development in the field of IPV (Brown & Hegarty, 2021; Ford-Gilboe et al., 2016; Hegarty et al., 2005). We undertook four steps: (1) generation and refinement of questionnaire items; (2) exploring face and content validity with experts; (3) embedding the IPSVS in a large, national survey of victim/survivors of IPV for psychometric testing; and (4) selection of final items. Steps 2 and 3 received ethics approval from The University of Melbourne Human Research Ethics Committee #21572 and #22680).

### Item Generation and Refinement

The IPSVS is based on a robust program of in-depth qualitative research on IPSV undertaken by the first author (Tarzia, 2021a, 2021b, 2021c; Tarzia & Tyler, 2021; Tarzia & Hegarty, 2022). This research involved detailed accounts from 38 women who had experienced IPSV. The transcripts from this study were carefully reviewed and behaviors reported by the women were compiled into an initial list of 33 preliminary items. Items spanned the continuum of severity in terms of the involvement of physical violence and harm (see Table 1).

**Table 1.** Initial List of Items and Revised Items.

Initial List of Items	Revised List of Items
1. Partner forced you to look at sexual images or watch pornography when it made you uncomfortable.	1. Made me look at sexual images or watch pornography when I did not want to.
2. Partner forced you to participate in sexual photos or videos without your consent.	2. Made me participate in sexual images or videos or took sexual images of me without my knowledge.
3. Partner forced you to act out something during sex that they had seen in sexual images or pornography.	Removed.
4. Partner made hurtful or abusive comments about your sexuality or sexual attractiveness (e.g., called you “frigid” or “ugly”).	3. Called me “frigid” or said I was sexually unattractive.
5. Partner made you perform sexual acts (or performed sexual acts on you) for the purpose of degrading or humiliating you.	4. Made me participate in sex acts or sexual positions that made me feel degraded.
6. Partner spoke to you in a degrading way during sex (e.g., called you names like “slut” or “whore”) when you did not consent to it.	5. Spoke to me in a degrading way during sex (e.g., called me names like “slut” or “whore”) when I did not consent to it.
7. Partner told you there was something wrong with you because you did not like what they were doing during sex.	Removed.
8. Partner compared you unfavorably to past sexual partners because you did not want to do something sexual.	6. Compared me unfavorably to past sexual partners.
9. Partner accused you of cheating because you did not want to have sex with them.	7. Accused me of cheating or not loving them when I said “No” to sex.
10. Partner accused you of not loving them if you refused to have sex with them.	
11. Partner pressured or badgered you for sex even after you said “No” or demonstrated disinterest.	8. Insisted that I have sex even after I said “No” or demonstrated disinterest.
12. Partner sulked or “froze you out” because you refused to have sex.	9. Sulked or “froze me out” because I refused to have sex
13. Partner verbally abused you if you refused to have sex.	10. Verbally abused me when I refused to have sex.
14. Partner made you have sex by threatening that something bad would happen if you said “No” (e.g., end the relationship, not give you money, throw you out of the house”).	11. Made me have sex by threatening that something bad would happen if I said “No” (e.g., end the relationship, not give me money, throw me out of the house).
15. Partner told you it was your duty or obligation to have sex with them.	12. Told me it was my duty or obligation to have sex with them.
16. Partner told you it was their right to have sex with you.	Removed.
17. Partner threatened or implied they would go elsewhere for sex if you did not have sex with them.	13, Threatened or implied they would go elsewhere for sex if I did not have sex with them.
18. Partner continued having sex with you after you said “Stop” or expressed discomfort, pain, or distress.	14. Forced me to continue having sex or performing sex acts even though I was in discomfort or distress or asked to stop.
19. Partner forced you to continue having sex or performing sex acts until they were finished, even though you were in discomfort or distress.	
20. Partner had sex with you or put their fingers inside you when you were asleep.	15. Had sex with me or performed a sex act on me when I was asleep, drunk, passed out or unconscious.
21. Partner made you have sex by threatening to physically hurt you, your children, or pets.	16. Made me have sex or perform a sex act by threatening to physically hurt me, my children, someone close to me or a pet.
22. Partner used physical force to make you have sex when you did not consent to it (e.g., held or pinned you down, hit you).	17. Used physical force to make me have sex or perform a sex act when I did not consent to it (e.g., held or pinned me down, hit me).
23. Partner choked or strangled you during sex without consent.	18. Choked or strangled me during sex without consent.
24. Partner penetrated you with an object without your consent.	Removed.
25. Partner behaved in an aggressive or violent way during sex when you did not consent to it (e.g., hitting or slapping you, penetrating you so hard that it caused damage).	19. During sex, behaved in an aggressive or violent way when I did not consent to it (e.g., hit or slapped me, penetrated me so hard that it caused damage).
26. Partner threatened you with a weapon to make you have sex with them.	20. Used a weapon to make me have sex or perform a sex act when I did not consent to it.
27. Partner became angry because you did not orgasm or show pleasure during sex.	21. Became abusive because I did not orgasm or show pleasure during sex.
28. Partner removed a condom during sex without your consent.	22. Removed a condom during sex without my consent
29. Partner deliberately performed a sex act or forced you into a sexual position they knew you did not like.	23. Made me participate in sex acts or sexual positions that they knew I did not like.
30. Partner forced you to consume alcohol or substances in order to prevent you from resisting sex.	24. Pressured me to consume alcohol or drugs so they could have sex with me.
31. Partner forced you to have sex when you were medically advised not to (e.g., post-partum or after surgery).	25. Made me have sex when I was medically advised not to (e.g., after surgery or childbirth).
32. Partner forced you to have sex with other people (including for money).	26. Made me have sex with other people (including for money) when I did not want to.
33. Partner forced you to participate in group sex when you did not want to.	Removed.
*New items added*	27. Made negative comments to other people about my sexual performance.
	28. Made me perform a sex act with an animal.
	29. Made me have sex in front of my child/ren or other people (including online).
	30. Threatened to share my sexual orientation or gender identity publicly if I would not have sex with them.
	31. Distributed sexual images of me without my consent.
	32. Made me watch them having sex with someone else.
	33. Made me have sex when there was a risk of contracting a sexually transmitted infection.
	34. Touched me in a sexual way or performed a sex act on me without my consent when they were my caregiver for a disability or illness.

The initial list of items was then sent to a range of experts including academics working in the area of interpersonal violence, specialist service providers, clinicians, and lived experience experts. Participants were asked to review the items and provide feedback via an online questionnaire designed using the Qualtrics platform. The questionnaire asked participants to rate the relevance of each potential scale item to the concept of IPSV from 0 (not relevant) to 10 (very relevant). Participants were also asked to comment on (1) wording or clarity of items; (2) whether any items should be removed; and (3) whether any items were missing from the scale. Twenty-four expert participants responded to the questionnaire; 14 were specialist service providers, 9 were academics or researchers and 2 were clinicians or other healthcare providers (categories were not mutually exclusive). Two participants reported lived experience of violence. Twenty-two of the participants identified as female and one as male. The majority of participants (*n* = 16) were located in Australia or New Zealand, six in the United Kingdom, and one participant elsewhere (did not specify). One participant did not provide any information about their role or other demographics.

In addition to the stakeholder questionnaire, the draft questions were reviewed by five victim/survivors who belong to a lived experience research advisory panel connected with the authors’ university. The research team also undertook numerous discussions about the items. Based on the results of the survey, the feedback from the victim/survivor panel, and conversations among the research team, from the initial list of 33 items, we:

• removed 4 items,
• merged 6 similar items into 3 reworded items,
• added a further 8 items to the scale for a total of 34 items and
• reworded some items for improved clarity and changed all items to first-person (see Table 1).

### Data Collection

#### Participants

A nationally representative sample of English-speaking women aged 18 years plus living in Australia were recruited for participation in a survey conducted online between February 14, 2022 and April 5, 2022. Recruitment was conducted by an experienced commercial research company, iLink, from a panel of people registered for research studies. Participation was voluntary and information about the survey emphasized that it was sensitive and confidential. A participation honorarium included panel points (paid by iLink in voucher form) and a draw to win one of two iPads.

#### Procedure

Prospective participants were screened for recent experience of IPV and eligible for the study if they reported a partner or ex-partner, in the last 5 years, had made them feel afraid; controlled their day-to-day activities or put them down; made them feel isolated from family, friends, or other people; made them feel monitored, manipulated, or harassed; threatened to hurt them or others they cared about; hit, slapped, kicked, or otherwise physically hurt them; or pressured or attempted to pressure them into sexual activity they did not want. Participants who progressed to the full survey were asked about IPSV as well as other forms of abuse and violence, health, service use, and demographic information. The survey included optional guided self-care activities and support information and took approximately 30 min to complete.

Participants reported their experience of 34 sexual violence items by any partner or ex-partner (of a relationship lasting longer than 1 month) in the last 5 years (*yes/no*). Participants who answered “yes” were presented with a Likert-type scale and asked how often in the last 12-months each behavior had occurred (*Not in the past 12 months*, *Once*, *A few times*, *Monthly*, *Weekly*, *Daily/almost daily*).

### Analysis

Data were analyzed using IBM SPSS 29.0.0.0 for Windows. To establish the structure of IPSV, the reliability and validity of the new scale, and a scale of practical length, factor analysis was the preferred analytical approach (Groth-Marnat, 2009; Pett et al., 2003; Tabachnick & Fidell, 2013). After assessing for missing values and limitations (Pett et al., 2003; Tabachnick & Fidell, 2013), common factor analysis was employed to identify the underlying dimensions of the 34 items (Costello & Osborne, 2005; Fabrigar et al., 1999; Sarstedt & Mooi, 2019; Tabachnick & Fidell, 2013). Image factoring extraction with promax rotation was employed (Pett et al., 2003; Tabachnick & Fidell, 2013). For the purpose of the analysis, participants were considered to have experienced IPSV if they had experienced any of the 34 behaviors at least once in the last 12 months.

## Results

Screening items were answered by 3,512 people with 1,452 deemed ineligible to continue the study because they had not recently experienced IPV and 426 participants did not finish the survey. A total of 1,026 participants completed the full survey, and of these, 702 reported having experienced one or more of the 34 IPSV items during the last 12 months (see Table 2 for demographics).

**Table 2.** Participant Demographics and Characteristics (*n* = 702).

Demographic Categories	Frequency	Percent
Age (years)		
18–24	101	14.4
25–44	325	46.3
>45	276	39.3
Gender of partner^a^ (*n* = 478)		
Male	362	51.6
Female	115	16.4
Non-binary	1	0.1
State of residence		
New South Wales	207	29.5
Victoria	196	27.9
Queensland	152	21.7
Western Australia	60	8.5
South Australia	57	8.1
Tasmania	16	2.3
Australian Capital Territory	12	1.7
Northern Territory	2	0.3
Residential regionality		
Metropolitan	444	63.2
Regional	230	32.8
Not specified	28	4.0
Indigenous status		
Aboriginal	38	5.4
Torres Strait Islander	1	0.1
Aboriginal and Torres Strait Islander	7	1.0
Neither Aboriginal or Torres Strait Islander	656	93.4
Citizenship (*n* = 701)		
Australian	563	80.2
Not Australian	138	19.8
First language		
English	629	89.6
Other	73	10.4
Highest education level		
Degree or higher degree	221	31.5
Diploma or certificate	264	37.6
Year 12 or lower	217	30.9
Employment status		
Fulltime (35+ hrs per week)	215	30.6
Part-time (<35 hrs per week)	212	30.2
Not currently employed	275	39.2

a Not all participants were in a relationship.

### Assessing Limitations

Data for 702 participants were screened for errors and cleaned. There were no univariate outliers; however, anomaly testing identified 17 cases as unusual. Although several of these cases displayed similar responses across multiple items, they were not excluded due to the nature of the items and the plausibility of respondents reporting these patterns of behaviors.

The sample size of 702, generating a case/variable ratio of 20.7, satisfied the minimum requirement for factor analysis (Pett et al., 2003; Tabachnick & Fidell, 2013). Not uncommon in domestic violence research (Ryan, 2013), an absence of univariate normality was found (Pallant, 2011), as were departures from linearity (Tabachnick & Fidell, 2013). The non-critical nature of univariate non-normality to factor analyses when working with large samples, and linearity when reducing the number of items and examining correlational patterns (Tabachnick & Fidell, 2013), resulted in a decision to review non-transformed factor analyses and re-assess only if the solution was uninterpretable (Field, 2009; Oppong & Yao, 2016; Tabachnick & Fidell, 2013). Finally, neither multicollinearity nor singularity was present in the data set (Field, 2009; Tabachnick & Fidell, 2013).

Examination of the factorability of the 34 items was undertaken, 77.0% of the correlations between pairs of variables exceeded 0.3%, and 99.4% of all correlations were significant at *p* = .01. Inspection of the anti-image correlation matrix confirmed that more than 99% of the correlations were less than .1 and the Kaiser-Meyer-Olkin (KMO) measure of sampling adequacy was .956. The dataset was thus deemed suitable for factor analysis (Field, 2009; Kaiser, 1974; Pett et al., 2003; Tabachnick & Fidell, 2013).

### Construct Validity

Thirty-four items were factor analyzed for preliminary review, using multiple common factor analysis extraction methods (Pett et al., 2003; Tabachnick & Fidell, 2013). The most interpretable, unrotated image factoring solution was selected for further exploration. After assessing scree plots, cumulative percentage of variances, and eigenvalues greater than 1.00, a decision to further examine the three- and four-factor solutions was made (Pett et al., 2003; Tabachnick & Fidell, 2013). Oblique rotation solutions were applied to both solutions (Pett et al., 2003; Tabachnick & Fidell, 2013). After considering matters of interpretability, meaning, and scientific utility (Pett et al., 2003; Tabachnick & Fidell, 2013), the forced three-factor solution with promax rotation was selected and retained.

Brevity of scale length was considered important due to the sensitive nature of IPSV, so lower-loading items were assessed for potential removal. Of the 15 items that loaded <.49 on their respective factors, 14 were removed after assessing their removal as inconsequential to the scale’s reliability (see Table 3). The one remaining low-loading item was considered conceptually important (Douglas et al., 2024) and thus retained (*Choked or strangled me during sex without consent*), resulting in a final scale of 20 items.

**Table 3.** Items Loading <.49 Removed from Scale.

Removed a condom during sex without my consent Made me have sex when there was a risk of contracting a sexually transmitted infection Made me have sex by threatening that something bad would happen if I said “No” Made me look at sexual images or watch pornography when I did not want to Made me participate in sexual images or videos, or took sexual images of me without my knowledge Pressured me to consume alcohol or drugs so they could have sex with me Called me “frigid” or said I was sexually unattractive Compared me unfavorably to past sexual partners Became abusive because I did not orgasm or show pleasure during sex Sulked or “froze me out” because I refused to have sex Spoke to me in a degrading way during sex when I did not consent to it During sex, behaved in an aggressive or violent way when I did not consent to it Used physical force to make me have sex or perform a sex act when I did not consent to it Had sex with me or performed a sex act on me when I was asleep, drunk, passed out, or unconscious

From a utility perspective, a scale containing 20 items was considered a suitable and practical length, so the solution was run a final time, revealing 3 clear factors. In this final solution, the second and third factors exchanged positions in the factor list, but the items continued to load consistently with their original factors. For naming purposes, the final factors were reviewed for common themes and with reference to the literature. Factor 1 (11 items; 35.369% of variance) was named “Dominance and Humiliation,” Factor 2 (5 items; 11.262% of variance) was named “Emotional Coercion,” and Factor 3 (4 items; 6.150% of variance) was named “Aggressive Indifference.” The final items and factor groupings, including their Cronbach’s alphas and explained variances, are shown in Table 4. Total variance explained by the items was 52.681%, considered acceptable in social science research (Pett et al., 2003).

**Table 4.** Summary of Final Three-Factor Solution with Reliabilities and Explained Variance.

Factor, Factor Name, Cronbach’s Alpha, Explained Variance, and Items	Loading
Factor 1: dominance and humiliation, α = .890 variance explained = 35.269% (11 items)	
Made me perform a sex act with an animal	.719
Made me have sex in front of my child/ren or other people (including online)	.678
Threatened to share my sexual orientation or gender identity publicly if I would not have sex with them	.675
Made me have sex with other people (including for money) when I did not want to	.674
Made me have sex or perform a sex act by threatening to physically hurt me, my children, someone close to me or a pet	.654
Made me watch them having sex with someone else	.652
Used a weapon to make me have sex or perform a sex act when I did not consent to it	.647
Distributed sexual images of me without my consent	.533
Touched me in a sexual way or performed a sex act on me without my consent when they were my caregiver for a disability or illness	.517
Made me have sex when I was medically advised not to (e.g., after surgery or childbirth)	.468
Choked or strangled me during sex without consent	.390
Factor 2: Emotional coercion, α = .741 variance explained = 11.262% (5 items)	
Accused me of cheating or not loving them when I said “No” to sex	.554
Verbally abused me when I refused to have sex	.504
Threatened or implied they would go elsewhere for sex if I did not have sex with them	.501
Told me it was my duty or obligation to have sex with them	.449
Made negative comments to other people about my sexual performance	.349
Factor 3: aggressive indifference, α = .785 variance explained = 6.150% (4 items)	
Made me participate in sex acts or sexual positions that they knew I did not like	.691
Made me participate in sex acts or sexual positions that made me feel degraded	.592
Forced me to continue having sex or performing sex acts even though I was in discomfort or distress or asked to stop	.544
Insisted that I have sex even after I said “No” or demonstrated disinterest	.459

*Note*. Image factoring extraction method, promax rotation method.

### Internal Consistency

The scale produced an overall internal consistency of α = .890. Cronbach’s alpha’s for the individual factors ranged from .741 to .890 (see Table 4). The internal consistency overall and of each set of items is considered acceptable (Devellis, 2017; Nunnally & Bernstein, 1994; Tavakol & Dennick, 2011).

## Discussion

The present study sought to develop a new survey instrument that was conceptually aligned with victim/survivors’ lived experiences of IPSV, was behaviorally based (Koss, 2007), comprehensive, and multi-dimensional. As discussed earlier, existing measures have not adequately captured the context and dynamics of IPSV as described in qualitative research (Bergen, 1995; Tarzia, 2021a, 2021b; Tarzia & Hegarty, 2022; Finkelhor & Yllo, 1985; Russell, 1990). In particular, the central role of degradation and humiliation (Tarzia, 2021a) and the use of sex as a tool to exert psychological dominance over the victim/survivor (Tarzia & Hegarty, 2022) have been largely overlooked in measurement of IPSV in favor of an emphasis on physical force.

The strength of this measure is that the IPSVS has face validity being based on in-depth accounts from victim/survivors (Tarzia, 2021a, 2021b, 2021c;Tarzia & Tyler, 2021; Tarzia & Hegarty 2022) and included a broad range of behaviors commonly reported in the literature (Bagwell-Gray et al., 2015; Barker et al., 2019; Tarzia, 2021a; Tarzia & Hegarty, 2022; Jeffrey & Barata, 2017; McOrmond-Plummer et al., 2014; Russell, 1990). The initial raft of questions underwent a robust review process with local and international stakeholders, lived experience experts, researchers, and those working in specialist services to ensure face and content validity. Data were collected from over 700 victim/survivors of IPSV via a national online survey, which was sufficiently large for psychometric testing. These steps are in line with best practice recommendations for scale development (Boateng et al., 2018; Carpernter, 2018; Devellis, 2017).

Factor analysis revealed a three-factor solution with acceptable internal reliability (Devellis, 2017; Tavakol & Dennick, 2011). The first factor, *Dominance and humiliation* (11 items), describes perpetrator tactics that aim to exert psychological dominance over the victim/survivor or to humiliate and degrade them. Unsurprisingly, the more extreme and severely physically violent items (e.g., “Choked or strangled me during sex without my consent,” “Made me perform a sexual act with an animal”) loaded onto this factor, but so did several items involving no or minimal physical contact. For instance, “Distributed sexual images of me without my consent” and “Made me watch them having sex with someone else.” This supports our earlier assertion that typologies focusing on the physical nature of sexually abusive behaviors are not optimal in terms of capturing the context and dynamics of IPSV. Rather, it is the intent behind the behaviors that is shared between the items in this factor—in this case, a desire to humiliate, shame, and exert psychological dominance over the victim/survivor (Tarzia & Hegarty, 2022).

The second factor, *Emotional coercion* (five items), describes verbal tactics that seek to promote guilt and insecurity in the victim/survivor. These included threats to look elsewhere for sex if the victim/survivor did not acquiesce, accusations of infidelity, and attempts to foster a sense of obligation in the victim/survivor. The use of emotionally manipulative behaviors to coerce a victim/survivor into unwanted sex is clearly described within the qualitative literature on IPSV (Tarzia, 2021c; Jeffrey & Barata, 2017; Johnstone, 2014; Russell, 1990) and captured within some of the existing sexual coercion scales (Raghavan et al., 2014). Survivors have reported feeling emotions such as guilt, self-blame, and anxiety about their relationships and a tendency to blame themselves for the IPSV, feelings that are strongly reinforced by the perpetrator (Tarzia, 2021c).

The third factor, which we have termed *Aggressive indifference* (four items), describes behaviors that can be viewed as an expression of the perpetrator’s complete disregard for the victim/survivor’s wellbeing in favor of their own sexual needs. Rather than seeking to dominate or humiliate the victim/survivor (as in the first factor), the perpetrator may be simply unconcerned about the victim/survivor’s discomfort or distress. For example, continuing to have sex after the victim/survivor has asked to stop, or insisting on sex even when the victim/survivor has said “No” loaded onto this factor, as did forcing the victim/survivor to perform sex acts they had made clear that they did not like or found degrading. Importantly, our findings distinguish between a victim/survivor being forced to participate in sex acts that they experience as degrading from the perpetrator’s specific intent to degrade their partner (as described in the first domain). It is essential to highlight that we have called this third domain “aggressive indifference” because the actions of the perpetrator go beyond simply being a “selfish lover.” Rather, the perpetrator may use sex as a way of conveying their utter disregard for the victim/survivor’s emotional and physical safety and wellbeing. Indeed, some victim/survivors have described feeling like “a piece of meat” or “empty and gross” upon the realization that their sexual autonomy was irrelevant to the perpetrator’s enjoyment (Tarzia, 2021a).

Suggested scoring for the IPSVS is that a “yes” answer to any one of the items would indicate that the respondent has experienced IPSV. While for some items, this is self-evident, for others, our decision could be viewed as being overly inclusive. For example, accusing someone of being unfaithful because they refused to have sex or making someone participate in sex acts they did not like is all too common in many relationships (Tarzia, 2021c). However, we feel that it is important to counteract societal normalization of such behaviors and foreground the significant harm that emotional manipulation, blackmail, and other forms of verbal coercion can do (Jeffrey & Barata, 2017; Logan et al., 2007; Tarzia et al., 2018) by labeling them as sexual violence.

### Future Directions and Limitations

We have collected data from over 700 women victim/survivors of IPSV as part of the development of this scale. Future research will explore the prevalence of different IPSV behaviors and its relationship with other forms of violence; in particular, how the different domains of the IPSVS align with physical and psychological violence and patterns of coercive control. This may lead to a more nuanced picture of IPSV that will have important implications for policy and best practice responses. It will also be critical to test the IPSVS with different populations to explore its applicability and acceptability.

The IPSVS addresses many of the current problems in measurement of IPSV; however, we also acknowledge several limitations, many of which relate to diversity. First, although the scale can be used with victim/survivors of any gender, the background qualitative work informing development of the IPSVS, and the development and validation itself, was undertaken entirely with women. Further exploration of the relevance of the IPSVS for men and gender-diverse victim/survivors is essential, since the context and dynamics of perpetration, and the types of behaviors, may differ. Similarly, although the proportion of Aboriginal and/or Torres Strait Islander women in our survey sample (6%) was higher than in the broader population (3.8%), and 10% of the participants did not have English as a first language, it is important to acknowledge that most of the research informing the development of the IPSVS was conducted with English-speaking, Australian-born victim/survivors who were not of Aboriginal or Torres Strait Islander background. Moreover, the applicability and relevance of the IPSVS to victim/survivors living in different cultural and socio-political contexts need to be determined through further research. In particular, the confronting nature of some of the questions may be problematic in some cultural contexts, where open conversations about sexuality and sexual violence are considered taboo (Tayyab & Sadaf, 2024). Finally, the IPSVS has undergone preliminary validation only. Further validation with a second sample is needed.

## Conclusion

The IPSVS is a comprehensive, multi-dimensional measure of IPSV that reflects the lived experiences of victim/survivors. Its domains highlight the critical role of perpetrator intent in understanding the complexity of IPSV and its different forms. We suggest that the IPSVS could be used to address the dearth of robust data on IPSV that is essential for developing strong policies and best practice responses.
